# Two Unexpected Tumors in a Laparoscopic Nephrectomy Specimen, Including a Rare Tubulocystic Renal-Cell Carcinoma: A Case Report

**DOI:** 10.1089/cren.2015.0021

**Published:** 2015-12-01

**Authors:** Jacob Jipp, Daniel Sadowski, Chad Defrain, Bradley Schwartz

**Affiliations:** ^1^ University of Iowa Carver College of Medicine, Iowa City, Iowa.; ^2^ Southern Illinois University School of Medicine, Division of Urology, Springfield, Illinois.; ^3^ Department of Pathology, HSHS St. John's Hospital, Springfield, Illinois.

## Abstract

We present a case of a 52-year-old Caucasian male who underwent a laparoscopic nephrectomy for an atrophic kidney and was found to have two unexpected, synchronous kidney cancers. He had a remote history of testicular cancer complicated by lymphadenopathy and external ureteral compression. Over time, he developed an atrophic left kidney from obstructive uropathy. Years later, due to flank pain and renal scintigraphy showing minimal function, a laparoscopic nephrectomy was performed. Final pathology demonstrated papillary renal-cell carcinoma (RCC) and tubulocystic RCC. Tubulocystic RCC is a rare neoplasm thought to be an indolent subset of collecting duct carcinoma, but was identified as a unique entity in 2004. Currently, there are ∼100 cases of this neoplasm in the literature.

## Introduction

Tubulocystic renal-cell carcinoma (RCC) is a rare tumor of the kidney that has recently been recognized as its own pathologic entity. Previously, it was thought to be a low-grade indolent subset of collecting duct carcinoma and was not separately discussed in the 2004 World Health Organization classifications. Amin et al. were the first to formally identify tubulocystic RCC as a unique cancer in a series of 31 subjects; this is the largest series to date.^1^ The remainder of the literature comprised either small series or case reports. In fact, there are still only ∼100 cases reported to date.^2^

In this study, we report a unique case of tubulocystic RCC. This tumor, along with the papillary RCC that was identified, was an unexpected finding in the nephrectomy specimen of an atrophic left kidney removed by minimally invasive surgery.

## Case Presentation

A 52-year-old male presented to our clinic for evaluation of an atrophic left kidney and left flank pain. He had a remote history of testicular cancer (type unspecified) in 2005, which was treated with orchiectomy, followed by chemotherapy. He had significant lymphadenopathy, which caused external compression of the left ureter. Massive hydronephrosis subsequently developed and stenting of the left ureter was attempted, but failed. Due to financial obstacles, he was then lost to follow-up for 10 years.

**FIG. 1.**
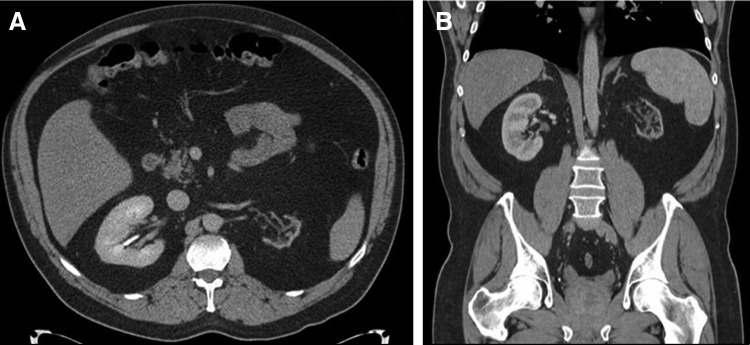
Axial **(A)** and coronal **(B)** CT slices demonstrating an atrophic *left* kidney without any identifiable mass or lesion.

He presented to his primary care provider with a chief complaint of urinary urgency and frequency in June 2015. As part of the work-up for his lower urinary tract symptoms, a renal ultrasound was obtained, which did not visualize the left kidney well and was equivocal for a left renal mass. A CT scan of the abdomen and pelvis with and without contrast then demonstrated a markedly atrophic left kidney without any excretion of the contrast material into the collecting system (Fig. 1). There was no hydronephrosis, perinephric stranding, or renal mass identified. After thorough discussion of the risks, benefits, and alternatives, he elected to proceed with left laparoscopic nephrectomy.

Gross pathology of the kidney showed a weight of 92 g and dimensions of 10 × 4 × 1 cm. There was a 1.2 × 0.9 × 0.9 cm orange–yellow mass identified in the inferior pole that bulged from the surface. Additionally, a 0.8 cm gelatinous white–tan nodule was identified in the superior pole contained within the renal capsule.

**FIG. 2.**
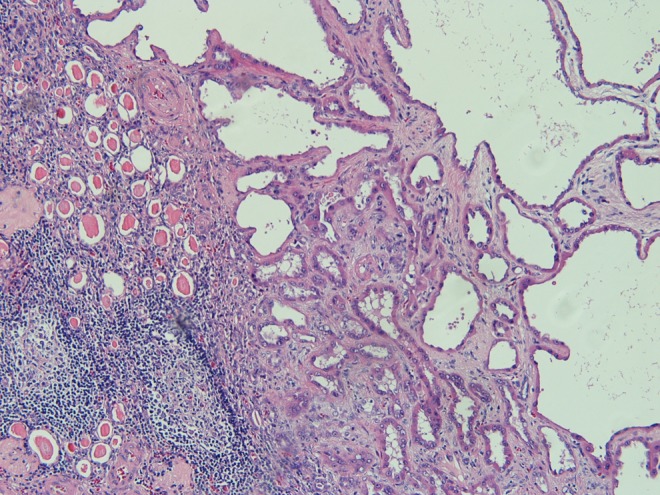
Low-power microscopy demonstrates cystically dilated tubules within a fibrotic stroma. Thyroidization and lymphoid follicles are noted in the surrounding atrophic renal parenchyma.

**FIG. 3.**
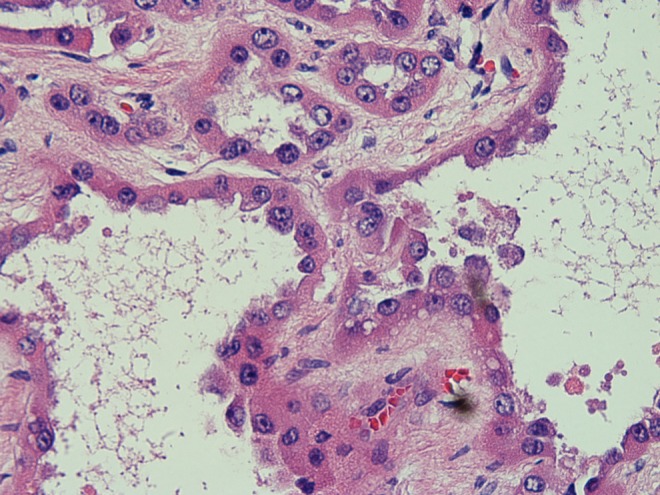
There is a hobnail appearance to the lining of the tubules characteristic of tubulocystic renal-cell carcinoma. There are prominent nucleoli and abundant cytoplasm in these neoplastic cells.

Microscopic analysis of the 1.2 cm inferior pole mass demonstrated a papillary RCC, Fuhrman grade II/IV. Focal tumor extension into the perinephric tissue was identified, but the margins were uninvolved. The final pathologic staging of this tumor was pT3a pNx. The superior pole mass was analyzed and identified as a tubulocystic RCC, Fuhrman nuclear grade III/IV (Figs. 2 and 3). Microscopic analysis did not reveal any tumor extension or positive margins. The final pathologic staging of this tumor was pT1a pNx.

Medical oncology was consulted for evaluation and review of therapy options for his papillary RCC. The nephrectomy has been deemed adequate treatment of the tubulocystic RCC.

## Discussion

To our knowledge, this is the first description of an unexpected finding of both papillary RCC and synchronous tubulocystic RCC in an atrophic kidney thought to be benign. Our patient fits the typical presentation of those with tubulocystic RCC. The majority of cases are identified in men with a mean age of 54 years.^1^ Similar to this case report, as much as 60% of the instances of tubulocystic RCC are identified in the left kidney.^1,3^ The majority of the cases in the literature are small tumors (pT1), and only rarely has there been evidence of metastatic disease.^1,3,4^

For decades, tubulocystic RCC was thought to be a subset of the typically aggressive collecting duct carcinoma of the kidney.^1^ These tumors were often identified as “Bellinien epitheliomas” or “carcinomas of the Bellini duct,” as they were suspected to originate from the collecting ducts of Bellini.^5^ The first reports of a supposed low-grade subset of collecting duct carcinoma arose in the 1990s. In 1997, Maclennan and colleagues published a series of 13 patients and posited that these tumors represented the low-grade indolent end of the collecting duct carcinoma spectrum.^5^ It was not until 2004 that Amin et al. presented a series of 31 patients and suggested the use of “tubulocystic carcinoma” to identify these tumors.^1^ Since then, Maclennan has revisited their series and reported that 8 of their 13 patients fit this description.^6^

Unique to this case report is the identification of synchronous papillary RCC in the nephrectomy specimen. There has been one previously reported case of tubulocystic RCC occurring simultaneously with clear cell RCC and micropapillary urothethial carcinoma of the bladder.^7^

## Conclusion

This case is the first description of a patient developing tubulocystic RCC in this capacity. The patient had a remote history of testicular cancer that caused obstructive lymphadenopathy. This resulted in the atrophic left kidney, but there is no evidence to suggest it had any impact on the development of the tubulocystic RCC. We believe the nephrectomy provided definitive treatment for the tubulocystic RCC, and the patient has been referred to an oncologist for further management of the papillary RCC given its higher stage.

## Abbreviations Used

CT: computed tomography
RCC: renal-cell carcinoma
